# Wernicke Encephalopathy in an Elderly Patient Due to Chronic Malnutrition From an Atypical Diet

**DOI:** 10.7759/cureus.14210

**Published:** 2021-03-31

**Authors:** Farhan A Shah, Shaidy Moronta, Michalla Braford, Priscilla Y Fujikawa, Gary Stocker

**Affiliations:** 1 Internal Medicine, Lewis Gale Medical Center, Salem, USA; 2 Internal Medicine, Edward Via College of Osteopathic Medicine, Blacksburg, USA; 3 Internal Medicine, Edward Via College of Osteopathic Medicine, Salem, USA; 4 Psychiatry, Lewis Gale Medical Center, Salem, USA

**Keywords:** wernicke encephalopathy, non-alcoholic wernicke's encephalopathy

## Abstract

Wernicke encephalopathy has traditionally been associated with chronic alcohol abuse leading to thiamine deficiency. Clinical symptoms include mentation change, gait ataxia, and oculomotor abnormalities. However, it is often an underdiagnosed condition in patients suffering from chronic malnutrition, especially in the West. We examine a unique case of non-alcoholic Wernicke encephalopathy in an elderly patient.

The patient had a long history of chronic malnutrition due to her atypical diet, consuming an unbalanced diet deprived of thiamine, unbeknownst to her. She presented with symptoms of encephalopathy, recurrent falls, and pupillary changes. After exhausting all other therapeutic interventions, she received a thiamine infusion; her mentation and other symptoms improved dramatically.

Thiamine deficiency can lead to severe complications, including Wernicke encephalopathy and cardiomyopathy. Wernicke encephalopathy can progress to Korsakoff syndrome, which is characterized by amnesia and confabulation. Case reports, such as ours, may remind clinicians to keep thiamine deficiency as a viable differential while evaluating acute encephalopathy, especially in the malnourished geriatric population.

## Introduction

Wernicke encephalopathy (WE) is a devastating disease characterized by the classic triad of acute mental status change, gait ataxia, and oculomotor dysfunction [1]. It is caused by thiamine (Vitamin B₁) deficiency. While WE is most commonly caused by chronic alcohol abuse, it can also be caused by chronic malnutrition in nonalcoholic patients. We present a case of WE in an elderly patient who had undiagnosed thiamine deficiency due to her chronic atypical diet.

## Case presentation

The patient is a 75-year-old Caucasian female with a past medical history of major depressive disorder who presented to the emergency department for altered mental status. She was brought in by ambulance after the patient developed acute aphasia and was unresponsive to verbal commands. She had developed generalized weakness, decreased oral intake, and recurrent falls over the past several weeks. Over the previous year, she had been increasingly developing abnormal dietary habits, including obsessively eating canned peaches, dairy products such as milk and cheese, and refusing to eat any leafy green vegetables. However, her mentation remained sharp, as she was able to have meaningful and in-depth conversations, up to until her recent symptoms had developed. Her initial vital signs indicated a temperature of 86.7 degrees Fahrenheit via rectal thermometer, hypertensive at 154/60 mmHg, bradycardic at 48 beats/minute, respiratory rate of 18 breaths/minute, and saturating 99% on room air. On physical exam, she was responsive only to painful stimulation, with a Glasgow Coma Score (GCS) of 8. However, she was able to spontaneously move all of her extremities and intermittently opened her eyes. Initial laboratory values were significant for hyponatremia of 120 mmol/L (reference range 135 - 145 mmol/L), glucose of 69 mg/dL (reference range of 74 - 106 mg/dL), white blood cell (WBC) count of 1.22 1000/uL (reference range of 4.50 - 10.50 1000/uL), ammonia was within normal limits as was the thyroid-stimulating hormone (TSH) (Table 1).

**Table 1:** Laboratory tests performed during hospitalization --- Test not performed on that day of hospitalization WBC: white blood cells; MCV: mean corpuscular volume; Plt: platelets; TSH: thyroid-stimulating hormone; Hct: hematocrit; Hgb: hemoglobin; T4: thyroxine; ESR: erythrocyte sedimentation rate

Test (reference range)	Day 1	Day 2	Day 3	Day 4	Day 5	Day 6	Day 7	Day 8	Day 9	Day 10	Day 11	Day 12	Day 13	Day 14	Day 15
WBC (4.50-10.50 x10*3/uL)	1.22	1.62	3.41	4.04	4.54	10.08	6.08	4.5	5.67	3.88	4.12	--	--	--	--
Hct (37.0-47.0%)	41	35	32.4	29.8	29.5	31.4	27.4	26.8	28	25	--	--	--	--	--
Hgb (11.4-15.5 g/dL)	14.1	12.4	11.3	10.1	10.1	10.6	9.1	8.7	8.8	8.5	7.4	7.7	7.8	8.2	8.1
MCV (80.0-100.0 fl)	84.4	83.1	84.4	84.4	85	85.8	88.1	89	90.6	89.9	90.2	--	--	--	--
Plt (130-385x10*3/uL)	53	47	63	48	52	98	118	150	210	282	332	--	--	--	--
Sodium (135-145 mmol/L)	120	127	130	130	131	132	135	138	138	137	139	138	142	141	141
Potassium (3.6-5.2 mmol/L)	3.8	4.1	3.7	3.6	4.1	4.2	4	3.7	3.6	3.6	3.3	3.1	3.1	3.8	3.7
Glucose (74-106 mg/dL)	69	90	69	110	104	75	100	90	117	96	77	98	84	75	97
Insulin (2.6-24.9 uIU/mL)	--	--	--	--	--	--	--	--	--	16.9	--	--	--	--	--
B12 (254-1320 pg/mL)	--	--	>6000	--	--	--	--	>3560	--	--	--	--	--	--	--
Thiamine (66.5-200.0 nmol/L)	--	--	--	--	--	--	217.9	214.9	196.7	--	230.8	--	225.2	--	163.6
Total Protein (6.4-8.2 g/dL)	6.7	--	--	5.3	--	--	--	5.4	5.4	--	--	--	--	--	--
Albumin (3.4-5.0 g/dL)	2.5	--	--	1.7	--	--	--	1.7	1.8	--	--	--	--	--	--
TSH (0.358-3.740 uIU/mL)	3.25	--	--	--	--	--	6.1	--	--	--	--	--	--	--	--
Free T4 (0.76-1.46 ng/dL)	--	--	--	--	--	--	--	0.96	--	--	--	--	--	--	--
ESR (0-20 mm/HR)	--	--	--	--	--	--	--	--	--	--	--	60	--	--	--

**Figure 1:**
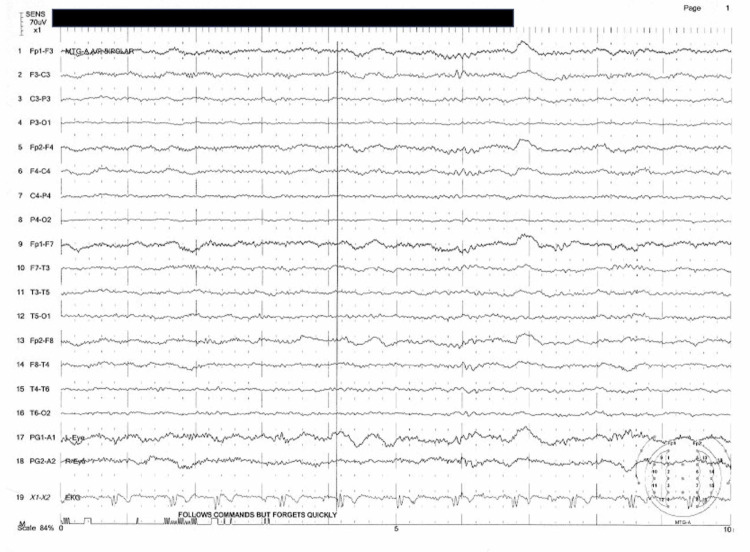
Electroencephalogram (EEG) revealed continuous generalized slowing that was suggestive of moderate encephalopathy

Initial CT scan of the head revealed a questionable hypodensity in the left middle cerebral artery (MCA) distribution in the temporal region. She was started on a forced-air warming blanket and admitted to the intensive care unit for concern for a cerebral vascular accident and hypothermia. Subsequent MRI of the brain revealed no acute intracranial findings, including no acute infarct. Mild fluid-attenuated inversion recovery (FLAIR) hyperintense foci within the supratentorial white matter was noted. Electroencephalogram (EEG) revealed continuous generalized slowing that was suggestive of moderate encephalopathy (Figure 1).

Lumbar puncture revealed a mildly elevated cerebrospinal fluid total protein of 53 mg/dL (reference range of 15-45 mg/dL) and glucose of 71 mg/dL (reference range of 40-70 mg/dL). Her hypothermia resolved and she was transferred to the general medicine wards, however, her mentation did not improve. She remained unresponsive to verbal commands, responded only to painful stimuli, and appeared to develop nuchal rigidity, with a flat affect. Lorazepam was administered to treat for possible catatonia, however, her symptoms did not improve. Given her previous history of malnutrition, she was administered intravenous high-dose thiamine (Vitamin B₁). The following day, the patient’s mentation had improved significantly. She was able to answer questions and hold conversations. She still had partial confusion, but was alert and oriented to person and time. Her family confirmed that her improvement was closer to how she was behaving prior to admission. Subsequently, she suffered a series of mechanical falls as a consequence of persistent gait ataxia. Following her falls, her level of alertness and speed of cognition continued to improve. As her cognition returned she began to question the daily plans told to her by her physicians, physical therapists, and even her family. While some of what she said included confabulation, she rapidly became more oriented to the events around her. By the time she was deemed stable for discharge, she was able to clearly explain why she was in the hospital, as well as her post-discharge plan, but she was not able to recall events that took place during the three weeks leading up to her admission. Afterward, she was discharged home.

## Discussion

The classic triad of WE consisting of altered mentation, ataxic gait, and sudden paralysis of ocular movement was first described in 1881 by Carl Wernicke. A similar condition affecting mentation, particularly in the creation of new memories, with subsequent polyneuropathy called Korsakoff syndrome (KS), was elucidated to be ‘two facets of the same disease’ by Russian psychiatrist SS Korsakoff between 1887 and 1891 [1]. It has been postulated that a spectrum exists between Wernicke encephalopathy and Korsakoff Syndrome (WKS), with WE being the initial reversible presentation of this disorder.

The clinical triad of altered mental status, gait ataxia, and ophthalmoplegia was found in only 16-33% of cases, with the most common sign being altered mental status in both alcoholic and non-alcoholic WE. Ocular findings can include nystagmus and conjugate gaze palsies due to the involvement of the oculomotor, abducens, and vestibular nuclei. The incidence of WE in the general population is approximately 0.4 to 2.8% compared to 12.5-35.0% in alcohol-dependent individuals [2,3,4]. Overall the prevalence ranges from 1 to 3% worldwide [5]. Mental status findings are broad and range from episodes of confusion and disorientation to coma and possible death. The disease is more prevalent in males (female to male ratio is 1:1.69) and no studies have demonstrated an increased incidence of WKS for any particular race. However, these statistics have largely been elucidated via postmortem studies illustrating the diagnostic challenge in recognizing WE, especially in non-alcoholic populations. Although classically associated with alcohol dependence, WKS can occur in a vast range of medical conditions including severe malnutrition, AIDS and other immunodeficiency syndromes, hyperemesis gravidarum, acute pancreatitis, inflammatory bowel disease, malignancy, thyrotoxicosis, liver disease, and as a consequence of bariatric surgery and total parenteral nutrition [5,6]. The epidemiology of non-alcoholic associated WKS differs from that of the alcoholic form with an increased incidence in females (1.84:1), younger age of onset, shorter duration of precipitating illness, higher association with ocular disturbances rather than gait disturbance, and favorable morbidity and mortality outcomes [2,4].

Thiamine deficiency is the primary cause of WKS and may also cause other conditions such as ‘Wet’ and ‘Dry’ Beriberi, which affect the cardiovascular and neurological systems, respectively [4,7]. Thiamine (Vitamin B₁ is a water-soluble vitamin that cannot be endogenously synthesized by humans and must be obtained via dietary sources such as beef, poultry, cereals, nuts, and beans. Therefore, conditions that predispose individuals to inadequate intake or malabsorption have been implicated in thiamine deficiency and the subsequent onset of WKS in a subset of these patients. Thiamine deficient and devoid states can deplete total body stores in as little as 18 days to up to 6 weeks [4,8]. In order to maintain total body thiamine reserves at 30-50 mg, the recommended daily intake of thiamine for adult men and women is 1.2 and 1.1 mg, respectively, and 1.4 mg during pregnancy and breastfeeding. Incidence of WE is higher in developing nations, likely due to increased malnutrition relative to resource access [5]. However, even calorically dense diets such as those provided by highly processed foods can be devoid of adequate amounts of thiamine and other nutrients [8]. Furthermore, overconsumption of foods rich in tannins, caffeine, theobromine, and theophylline (coffee, chocolate, and tea) can inactivate thiamine and further deplete body stores. Hence, we must be cognizant of micronutrient malnutrition even in patients that do not fall into the classic malnutrition picture.

Thiamine predominantly exists in its bioactive form thiamine pyrophosphate (TPP, also called thiamine diphosphate) before undergoing hydrolysis to free thiamine and absorption in the jejunum [4,7,8]. After carrier-mediated transport across intestinal epithelia, free thiamine may enter erythrocytes and undergo rephosphorylation to TPP via cytoplasmic thiamine pyrophosphokinase [7]. TPP serves as a cofactor in many biochemical processes within cells essential to cellular metabolism, energy production, and antioxidation [4,5,8].

**Figure 2:**
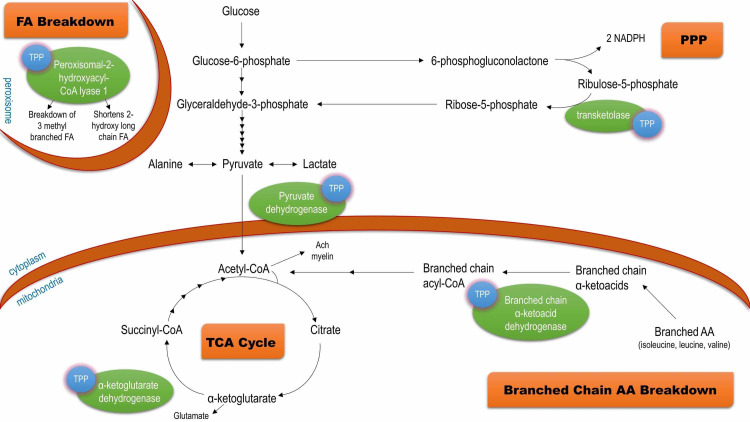
Thiamine pyrophosphate (TPP) dependent pathways including fatty acid (FA) breakdown, tricarboxylic acid (TCA) cycle, pentose phosphate pathway (PPP), and branched-chain amino acid (AA) breakdown NADPH: nicotinamide adenine dinucleotide phosphate

Thiamine is vital for aerobic respiration provided by the tricarboxylic acid (TCA) cycle (Figure 2). It is a cofactor for the enzyme pyruvate dehydrogenase which links anaerobic glycolysis to the TCA cycle and as a cofactor for α-ketoglutarate, ultimately produces cellular energy in the form of ATP [3,5]. Notably, with increased dietary carbohydrate intake, the demand for thiamine also increases as it is a key factor in glucose metabolism [8]. TPP also acts as a cofactor for transketolase in the non-oxidative branch of the pentose phosphate pathway (PPP). This pathway produces ribose 5-phosphate (R5P) and nicotinamide adenine dinucleotide phosphate (NADPH); products essential to nucleotide biosynthesis, free radical scavenging, and fatty acid synthesis (including myelin) [3,8]. The PPP is especially important for adequate cerebral functioning along heavily myelinated tracts and high proliferating tissues [8,9]. Thiamine also serves as a cofactor for peroxisomal 2-hydroxyacyl-CoA lyase 1 and branched-chain α-ketoacid dehydrogenase.

Depletion in thiamine results in reduced availability of ATP and NADPH, reduced levels of α-ketoglutarate, citrate, acetylcholine, and subsequent accumulation of toxic intermediates lactate, pyruvate, alanine, and glutamate. These factors may contribute to the neurologic impairment seen in WKS as the brain is heavily reliant on glucose metabolism for ATP and is vulnerable to local imbalances in pH as a result of toxic substrate accumulation [8]. Several studies have implicated neuronal cell death to distinctly affect the mamillary bodies and thalamus in WKS; lesions which have also been demonstrated on MRI and CT [5]. The disruption in cellular homeostasis also impacts the integrity of the blood-brain barrier (BBB) leading to vasogenic edema which may also be implicated in the onset of WKS [3]. Astrocytes maintain the tight junctions necessary for optimum functioning of the BBB; this role is directly disrupted by ATP depletion, oxidative stress, acidic conditions, and excess glutamate in the synaptic clefts leading to excitotoxicity and ultimate failure [3,4].

The diagnosis of WE is largely a clinical diagnosis following a thorough history and physical exam. A clinical diagnosis is typically made when at least two of the following criteria are met: eye signs, dietary deficiency of thiamine, altered mental status, or cerebellar dysfunction. Additional tests, including a complete blood count and comprehensive metabolic panel, can be conducted to exclude similar conditions that may mimic WE. MRI may uncover increased signaling in the periventricular thalamus, mammillary bodies, and periaqueductal gray matter. Erythrocyte transketolase is occasionally used to detect thiamine deficiency [5]. It has been stated that over-diagnosis coupled with over-treatment may be preferred in order to prevent the prolonged neurocognitive consequences of WE, given the high safety profile of thiamine [10]. 

Given the cause of WE is an underlying thiamine deficiency, treatment revolves around thiamine repletion. Administration of 100 mg of thiamine (IV [intravenous] or IM [intramuscular]) for 3-5 days is standard therapy. Occasionally, magnesium will need to be replaced as well using magnesium sulfate 2 grams IV every 6-8 hours or magnesium oxide 400-800 mg orally each day. Additionally, supportive therapy should be given as well including rehydration, nutritional therapy, and correcting for any electrolyte abnormalities [11].

Clinical manifestations of WE have varied responses to treatment. Apathy, confusion, and drowsiness respond favorably, whereas memory and learning deficits are longer lasting. Mental status changes along with acute encephalopathy gradually resolve. Oculomotor abnormalities respond quite well to treatment with horizontal and vertical gaze palsies completely resolving after several days to weeks. Horizontal nystagmus, however, may persist for several months in roughly 60% of patients. A full recovery is possible, but most individuals still experience difficulty with gait [3].

Approximately 75% of untreated WE turns into KS [12]. KS is characterized by global amnesia and anterograde/retrograde memory deficits. Episodic and semantic memories are commonly affected while implicit memory usually remains intact. Patients frequently confabulate and compensate for these gaps in memory by replacing them with untrue facts [13,14]. KS is accounted for by alcoholism in 95% of cases [15].

One controversial aspect of KS is the area responsible for memory impairment. Most affected structures are in the third and fourth periventricular regions. Previous studies showed that either the dorsomedial or the anterior thalamic nuclei could be contributing to deficits in memory seen in KS. More recent studies then revealed that damaged dorsomedial nuclei in a patient with intact anterior nuclei did not correlate with severe memory impairment. Therefore, we can conclude that dysfunction in the anterior thalamic nuclei is key to severe memory impairment and anterograde amnesia. Other areas affected include the dorsomedial thalamic nuclei, mammillary bodies, basal forebrain, dorsal and median raphe nuclei, and the cerebellar vermis. In cases of ethanol neurotoxicity, the superior frontal cortex is damaged leading to executive impairment. Alcohol-related KS is also associated with a decrease in gray and white matter volume. However, there are several reports of nonalcoholic KS in which no atrophy was seen on MRI. Therefore, although changes in periventricular areas are present in half of the cases of WE, and they can support a clinical suspicion of KS, diagnosis of KS should not rely solely on imaging [14,15,16].

Approximately 25% of patients who develop KS are permanently disabled. In cases of alcoholic KS, the key to long-term treatment is alcohol abstinence. Some patients with alcohol-related dementia were noted to improve on memantine therapy and reports have been published about the benefits of donepezil for memory improvement. However, this same benefit was not seen in cases of nonalcoholic KS. Therefore, pharmacologic therapy is limited and the best treatment approach includes a combination of both pharmacology and behavioral interventions, such as cognitive therapy and external aids [14].

## Conclusions

This case report highlights how insidious the presentation of non-alcohol-induced WE can be. Thiamine deficiency remains an underdiagnosed and undertreated vitamin deficiency throughout the globe, even in the Western world. Complications of the disease may involve multiple organ systems, such as the heart and brain. Clinicians need to remain cognizant that chronic malnutrition, especially in the geriatric population, can result in thiamine deficiency and subsequent WE. Physicians should continue to educate themselves and their patients accordingly.
